# Enhancing Third-Order Nonlinear Optical Property by Regulating Interaction between Zr_4_(embonate)_6_ Cage and N, N-Chelated Transition-Metal Cation

**DOI:** 10.3390/molecules28052301

**Published:** 2023-03-01

**Authors:** Gang Xiang, Na Li, Guang-Hui Chen, Qiao-Hong Li, Shu-Mei Chen, Yan-Ping He, Jian Zhang

**Affiliations:** 1 College of Chemistry, Fuzhou University, Fuzhou 350108, China; 2 State Key Laboratory of Structural Chemistry, Fujian Institute of Research on the Structure of Matter, Chinese Academy of Sciences, Fuzhou 350002, China

**Keywords:** zirconium-organic cages, self-assembly, structures, nonlinear optical

## Abstract

Herein, the combination of anionic Zr_4_L_6_ (L = embonate) cages and N, N-chelated transition-metal cations leads to a series of new cage-based architectures, including ion pair structures (**PTC-355** and **PTC-356**), dimer (**PTC-357**), and 3D frameworks (**PTC-358** and **PTC-359**). Structural analyses show that **PTC-358** exhibits a 2-fold interpenetrating framework with a 3,4-connected topology, and **PTC-359** shows a 2-fold interpenetrating framework with a 4-connected **dia** network. Both **PTC-358** and **PTC-359** can be stable in air and other common solvents at room temperature. The investigations of third-order nonlinear optical (NLO) properties indicate that these materials show different degrees of optical limiting effects. It is surprising that increasing coordination interactions between anion and cation moieties can effectively enhance their third-order NLO properties, which can be attributed to the formation of coordination bonds that facilitate charge transfer. In addition, the phase purity, UV-vis spectra, and photocurrent properties of these materials were also studied. This work provides new ideas for the construction of third-order NLO materials.

## 1. Introduction

In recent years, third-order NLO materials have already had widespread applications in many fields [1,2,3,4,5,6,7,8], such as optical switching, optical limiting, optical communications, optical data processing, and mode-locked laser system, etc. To date, multifarious elaborately designed organic/inorganic and hybrid materials have been synthesized for third-order NLO [9,10,11,12,13,14,15], including semiconductor quantum dots, black phosphorus, carbon nanodots, polymers or conjugated organic molecules (such as porphyrins and phthalocyanines), metal-clusters or metal-oxo clusters and metal-organic frameworks, and so on. It is acknowledged that ionic pair compounds are potentially excellent NLO materials because of their intrinsic attributes, such as high positive/negative charge, ordered self-assembly structures and stability [16,17,18,19,20], and the process of electron/charge transfer between anion and cation groups is usually considered as the significant factor for favorable NLO response [21,22,23,24]. With the enhancement of the covalent or coordinative interactions between anion and cation groups, the third-order NLO properties also increase, which may be because the covalent or coordinative interactions are beneficial to the electron/charge transfer between anion and cation groups [25,26].

Very recently, our group has been devoted to research work based on an anionic metal-organic cage (Ti_4_L_6_ or Zr_4_L_6_, L = embonate) [27]. It is an interesting dispersed molecular cage with a tetrahedral symmetry environment, and it can be soluble and stable in water and some common solvents. In the study, we found that it is an excellent building block for stepwise assembly due to its abundant interaction sites (uncoordinated carboxyl oxygen atoms and naphthyls), facilitating the incorporation of metal coordination bonding, π-π stacking, and other noncovalent interactions altogether. By using such ultra-stable cage as a reactive precursor to assembly with metal ions and highly conjugated organic ligands or groups [28,29,30], a few advanced cage-based materials with various dimensional structures were obtained by two-step reaction, including 3D frameworks with hydrocarbons sorption capacities, simple cage compounds with identification and separation functions, and a series of co-crystals with notable third-order NLO performances.

**Scheme 1:**
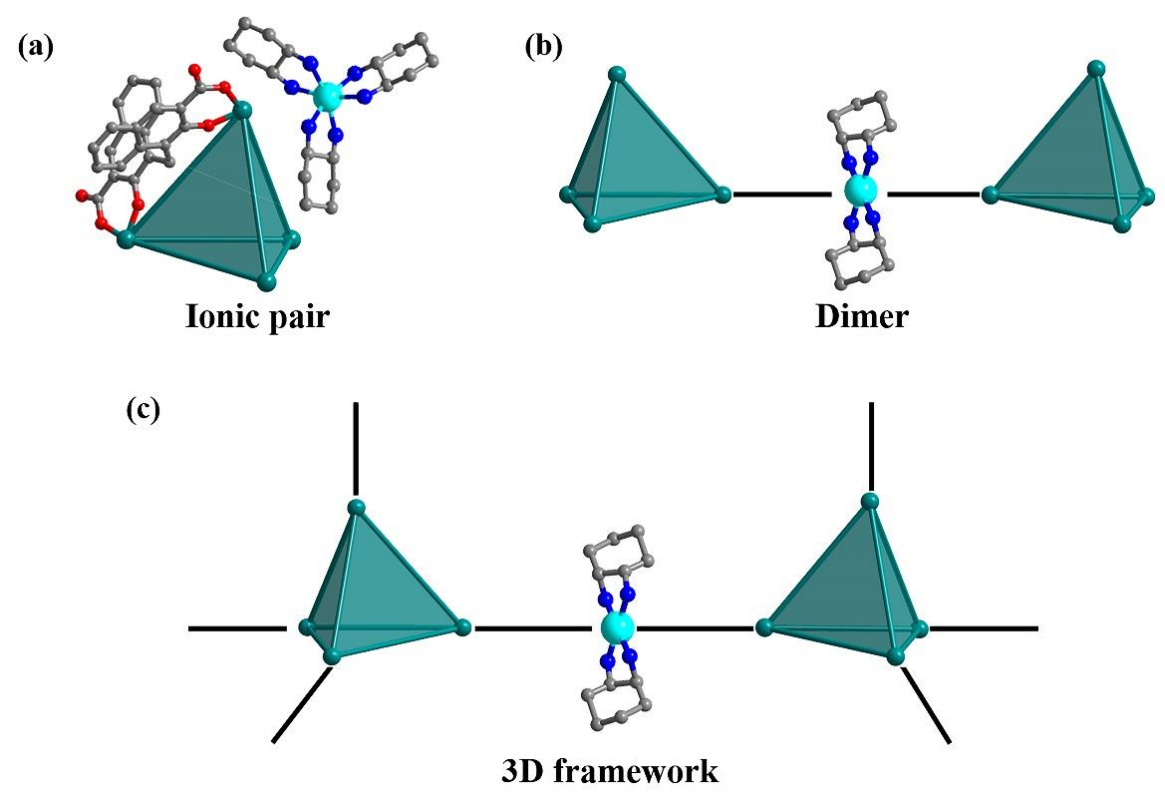
(**a**) Ionic pair. (**b**) Dimer. (**c**) 3D framework of the self-assemblies between Zr_4_L_6_ cage and N, N-chelated zinc cation.

In this work, we investigate the assembly behavior of anionic Zr_4_L_6_ cage and N, N-chelated transition-metal cations (Scheme 1), and we explore the effect of charge transfer between them on third-order NLO performances. More specifically, through changing the type and molar ratio of the salt and solvent, the reaction of Cu^+^ or Zn^2+^ ions, 1,2-diaminocyclohexane, and Zr_4_L_6_ cages produced a series of novel Zr_4_L_6_-based architectures, including ion pair structures (**PTC-355** and **PTC-356**), dimer (**PTC-357**), and 3D frameworks (**PTC-358** and **PTC-359**). X-ray single crystal structural analyses show that **PTC-358** exhibits a 2-fold interpenetrating framework with 3,4- connected topology, and **PTC-359** shows a 2-fold interpenetrating framework with a 4-connected **dia** network. Both **PTC-358** and **PTC-359** can be stable in air and other common solvents at room temperature. In addition, the phase purity, UV-vis spectra, and photocurrent properties of these materials were also studied. For these cage-assembled compounds **PTC-355**–**PTC-359**, their emission peaks can be observed at 460, 472, 490, 510, and 501 nm, respectively. Furthermore, by means of a film-making method, we tested the third-order NLO properties of these materials. We found that the efficiency of the charge transfer was greatly enhanced with the increasing coordinative interaction between the anion cage and cation group, leading to the enhancement of NLO response.

## 2. Results and Discussion

**Figure 1:**
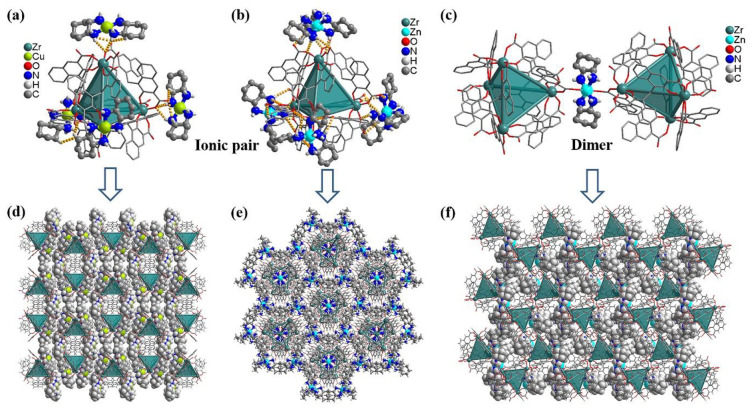
Two cage-based ionic pairs composed of a Zr_4_L_6_ cage and different metal-cation units: **PTC-355** (**a**) and **PTC-356** (**b**). (**c**) The dimer structure of **PTC-357** formed by two Zr_4_L_6_ cages and one [Zn(cis-DCH)2]^2+^ cation. (**d**–**f**) The 3D packed structures of **PTC-355**–**PTC-357**, respectively. Atom color code: green, Cu; powder blue, Zn; olive, Zr; red, O; blue, N; gray, C; off white, H. Some H atoms have been removed for clarity.

The simple method previously reported was used to synthesize large amounts of PTC-101(Zr) as the source of the Zr_4_L_6_ cages [21], which is also described in detail in the experimental section. In the beginning, CuBr and (+/−)-trans-1,2-diaminocyclohexane (*trans*-DCH) were added to the DMF/H_2_O solution of PTC-101(Zr), which was kept at room temperature for 3 days, yielding blue block crystals of **PTC-355**. Single-crystal structural analysis reveals that **PTC-355** crystallizes in a monoclinic system with space group *C*2/*c*, and the asymmetric unit contains half of anionic Zr_4_L_6_ cage, two [Cu(*trans*-DCH)_2_]^2+^ cations, and some solvent molecules, as shown in Figure S1 in the Supporting Information. In **PTC-355**, the Cu(II) center is chelated by two *trans*-DCH ligands (Figure 1a) and shows [CuN4] planar quadrilateral coordination geometry, giving rise to a [Cu(*trans*-DCH)_2_]^2+^ ion. The Cu-N bond distances vary from 1.96 to 2.03 Å. The calixarene-like oxygen vertices of each Zr_4_L_6_ cage match very well with four [Cu(*trans*-DCH)_2_]^2+^ units. Through weak C/N−H⋯O (2.9–3.4 Å) and C−H···π (3.6–4.0 Å) interactions, the Zr_4_L_6_ cages and the [Cu(*trans*-DCH)_2_]^2+^ cations stack alternately into a 3D dense supramolecular structure (Figure 1d and Figure S2). By replacing the above copper salt (CuBr) with zinc salt (Zn(NO_3_)_2_·7H_2_O) and changing the solvent type (we used 1,4-dioxane/H_2_O/MeCN as solvent), as well as raising the reactive temperature (80 °C), we successfully synthesized yellow block crystals of **PTC-356**. Single crystal structural analysis shows that **PTC-356** crystallizes in a trigonal system with space group *R*3*c*. In the asymmetric unit of **PTC-356**, there are one-third of the Zr_4_L_6_ cage and one and one-third of [Zn(*trans*-DCH)_3_]^2+^ cations (Figure S3). Unlike **PTC-355**, in the structure of **PTC-356**, the Zn(II) center in the [Zn(*trans*-DCH)_3_]^2+^ unit is chelated by three *trans*-DCH ligands (Figure 1b) and exhibits [ZnN6] octahedral coordination geometry. The Zn−N bond distances vary from 2.18 to 2.21 Å. Because of its highly symmetrical crystal system, **PTC-356** has an attractive honeycomb-like packing superstructure (Figure 1e). There is no typical hydrogen bond, and the binding interaction may be due to weak van der Waals force between zirconium cages and cation units. However, when *trans*-DCH and Zn(NO_3_)_2_·7H_2_O were replaced with *cis*-1,2-diaminocyclohexane (*cis*-DCH) and Zn(CH_3_COO)_2_·2H_2_O, respectively, compound **PTC-357** was obtained. Structural analysis displays that **PTC-357** crystallizes in a triclinic system with space group *P*-1, and the asymmetric unit includes one Zr_4_L_6_ cage, half of [Zn(*cis*-DCH)_2_]^2+^ and two [Zn(*cis*-DCH)_3_]^2+^ cations, and one (Me_2_NH_2_)^+^ cation. In the structure of **PTC-357**, an interesting dimer can be observed (Figure 1c), in which two Zr_4_L_6_ cages are connected by one [Zn(*cis*-DCH)_2_]^2+^ unit. Herein the Zn(II) center is six-coordinated by four N atoms from two *cis*-DCH and two carboxyl O atoms from two tetrahedrons (the Zn–O bond distance is 2.44 Å) to build a distorted tetrahedral coordination geometry. The Zn−O bond distances vary from 2.00 to 2.24 Å. These dimers and surrounding [Zn(*cis*-DCH)_3_]^2+^ cations are further packed into a 3D dense architecture (Figure 1f and Figure S5).

**Figure 2:**
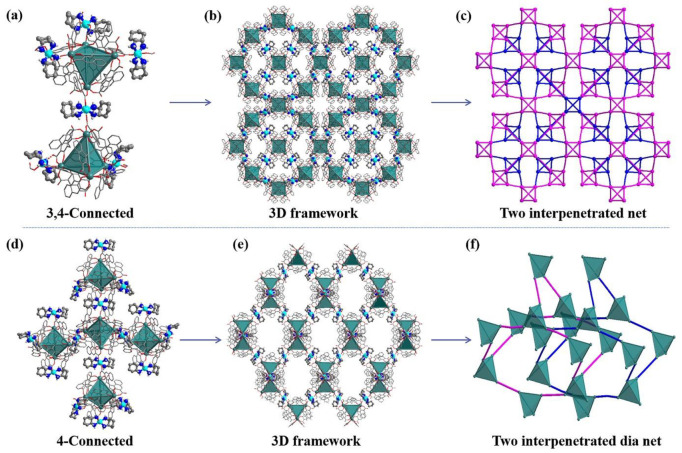
(**a**,**b**,**d**,**e**) The connectivity between Zr_4_L_6_ cages and [Zn(cis-DCH)2]^2+^ cation creates a 3D framework **PTC-358** and **PTC-359**, respectively, and their interpenetrated networks (**c**,**f**). Atom color code: green, Cu; powder blue, Zn; olive, Zr; red, O; blue, N; gray, C; off white, H. Some H atoms have been removed for clarity.

Encouraged by the above results, more synthetic experiments were explored. By replacing Zn(CH_3_COO)_2_·2H_2_O with ZnSO_4_·7H_2_O and reducing the amount of *cis*-DCH in the above synthetic procedure of **PTC-357**, **PTC-358** was prepared. Compound **PTC-358** has a highly symmetric cubic chiral space group *I*23, and its asymmetric unit possesses five out of six Zr_4_L_6_ cages and one [Zn(*cis*-DCH)_2_]^2+^ cation (Figure S6). Interestingly, such in situ generated [Zn(*cis*-DCH)_2_]^2+^ unit further connects two adjacent Zr_4_L_6_ cages through coordination bonds (one Zn–O bond distance is 2.197 Å and another is the weak force of 2.827 Å), and the Zr_4_L_6_ cages are coordinated by three or four [Zn(*cis*-DCH)_2_]^2+^ units (Figure 2a), giving rise to a 3D framework possessing large cavities (Figure 2b). The large intraframework spaces are occupied by another identical but independent framework, giving a 2-fold interpenetrating structure (Figure 2c). Considering the bridging [Zn(*cis*-DCH)_2_]^2+^ units, the Zr_4_L_6_ cages act as three- or four-connected nodes, so the network topology of **PTC-358** can be described as a (3,4)-connected net. However, by reducing the amount of Zn(CH_3_COO)_2_·2H_2_O and *cis*-DCH in the above synthetic procedure of **PTC-357**, we obtain compound **PTC-359**. Structural analysis shows that **PTC-359** crystallizes in a monoclinic system with space group *P*2_1_/*n* and the asymmetric unit involves two Zr_4_L_6_ cages, four [Zn(*cis*-DCH)_2_]_2_^+^ cations, and one (Me_2_NH_2_)^+^ cation. **PTC-359** exhibits a 4-connected **dia**-type network of Zr_4_L_6_ tetrahedra linked together by [Zn(*cis*-DCH)_2_]^2+^ units (Figure 2d,e). In **PTC-359**, a larger diamondoid cage is constructed by ten Zr_4_L_6_ cages and twelve [Zn(*cis*-DCH)_2_]^2+^ units (Figure 2f). Similarly, because the potential space is large enough to fill two independent frameworks, forming a 2-fold interpenetrated structure. This is a very interesting phenomenon in the field of supramolecular self-assembly, which also shows that the reaction conditions have a significant effect on the assembly behavior of titanium cage and zinc units.

**Figure 3:**
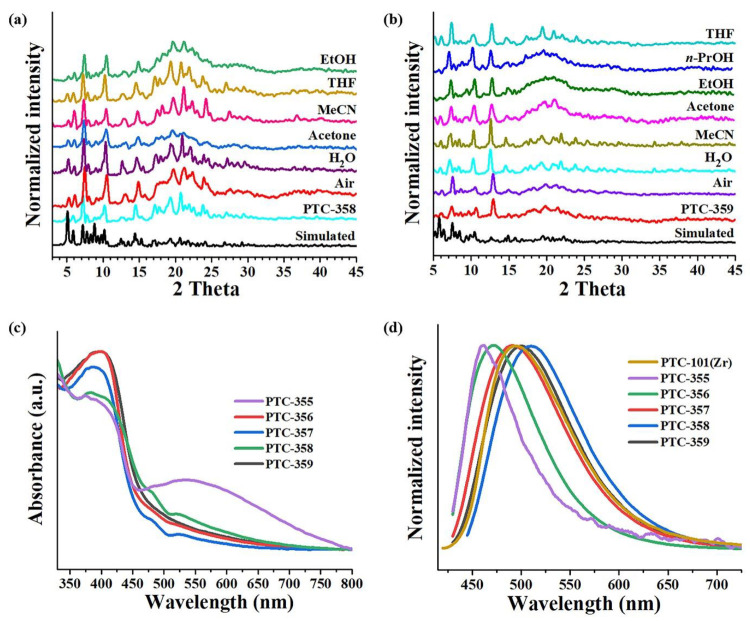
(**a**,**b**) The stability study for **PTC-358** and **PTC-359** (Some crystals were exposed to air for 12 h or immersed in different solvents for 1 day). (**c**) Solid-state UV/vis absorption spectra of **PTC-355**–**PTC-359**. (**d**) Normalized emission spectra measured in air at room temperature for PTC-101(Zr), compounds **PTC-355**–**PTC-359**.

The powder X-Ray Diffraction (XRD) of **PTC-355**–**PTC-359** confirmed the phase purities of these samples (Figure 3a,b and Figures S13–S15) because their XRD patterns are very similar to those simulated from their single crystal data. The above results show that the self-assembly of Zr_4_L_6_ cages with N, N-chelated zinc units into **PTC-356**–**PTC-359** under certain conditions is quite stable and directed. Thermal gravimetric analyses (TGA) curves of these materials were measured, as shown in Figures S8–12 in the Supporting Information. The TGA curve of **PTC-355** shows a distinct weightless platform before 250 °C. For other compounds, the solvents were gradually lost with increasing temperature, followed by the decomposition of their structures. The residues after decomposition may be ZrO_2_ and CuO or ZnO. In addition, the stabilities of **PTC-358** and **PTC-359** were also studied. At room temperature, some crystals of **PTC-358** and **PTC-359** were exposed to air for 12 h or immersed in different solvents for 1 day, respectively, and then their XRD patterns were examined. As shown in Figure 3a,b, the main peaks of their XRD patterns are maintained, although very individual peaks widen or disappear. Obviously, both of them can be stable in air, H_2_O, and other common solvents, such as acetonitrile (MeCN), ethanol (EtOH), tetrahydrofuran (THF) and acetone, etc. In contrast, **PTC-355** and **PTC-356** have poor air and solvent stabilities. Herein the coordination and interpenetrating frameworks have good stabilities compared to the supramolecular packing frameworks. Diffuse reflectance spectroscopy was used to study the UV/Vis absorption of these compounds. According to the Kubelka–Munk function, they exhibit relatively low bandgaps (Figure 3c). To understand the structure–function relationship, the electronic properties and frontier orbitals of **PTC-359** were calculated based on the density function theory (DFT). From Figure S26, the highest occupied molecular orbital (HOMO) is mainly provided by the embonate (L) ligand and Zr atom, and the lowest unoccupied molecular orbital (LUMO) is mainly provided by another L and [Zn(cis-DCH)_2_]^2+^ unit. The electron transfers between HOMO and LUMO are mainly ligand-to-ligand charge transition (LLCT) and a small part of metal-to-ligand charge transition (MLCT). Under the electrostatic interaction of anion Zr_4_L_6_ cage and [Zn(cis-DCH)_2_]^2+^ cationic unit, it is beneficial for the charge transfer from L in Zr_4_L_6_ cage to L combined with [Zn(cis-DCH)_2_]^2+^ unit, which may lead to good NLO performance.

We also studied the solid-state excitation and emission spectra of compounds **PTC-355**–**PTC-359** at room temperature. Since the π-conjugated L (embonate) ligand possesses a strong absorbing chromophore, the Zr_4_L_6_ cages themselves have good photoluminescent properties. As shown in Figure S16, upon excitation at 363 nm, the L ligand displays an emission band at 420–700 nm (Figure S17), which may belong to the π→π* transition. When excited at 430 nm, PTC-101(Zr) shows a similar emission peak near 495 nm (Figure 3d). For these cage-assembled compounds **PTC-355**–**PTC-359**, their emission peaks can be observed at 460, 472, 490, 510, and 501 nm, respectively. Their excitation spectra are shown in Figures S19–23. Remarkably, **PTC-355** and **PTC-356** exhibit significant blue shifts (ca. 20–40 nm) in comparison with PTC-101(Zr), and **PTC-358** displays a blue shift of 15 nm. In this case, the ligand-based luminescence is in a dominating place, and the blue-shifted or red-shifted phenomenon may be attributed to the metal–ligand/cage coordinative interactions [31,32].

**Figure 4:**
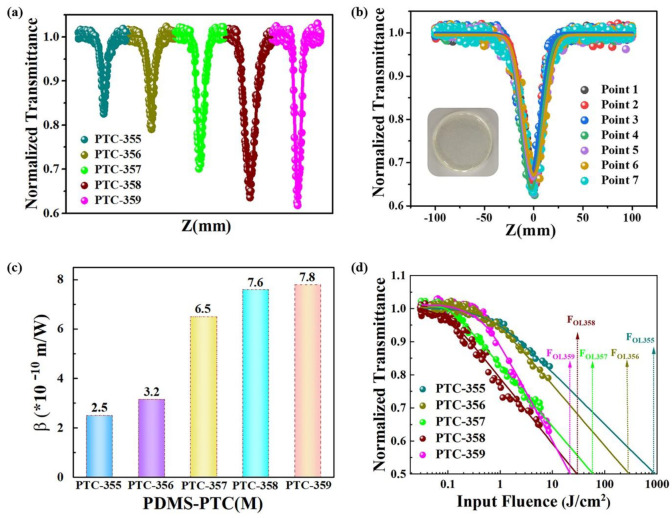
(**a**) OA Z-scan (points) and theoretical fit (solid lines) curves of **PDMS-PTC-355**–**PDMS-PTC-359** films at 532 nm. (**b**) The stability and repeatability of third-order NLO responses for the **PDMS-PTC-359** film at 532 nm (inset: photo of the **PDMS-PTC-359** film). (**c**) Comparison of nonlinear absorption coefficients (β) of PDMS-PTCs films. (**d**) Variation in the normalized transmittance as a function of input intensity.

Considering ionic pairs are potentially excellent nonlinear optical materials due to their intrinsic attributes, such as high positive/negative charge and ordered self-assembled structures, we decided to study the third-order NLO property of **PTC-355**–**PTC-359**. In order to ensure the stability of its structure and expand its practical optical applications, these crystal materials were evenly dispersed into PDMS (polydimethylsiloxane), and then flexible and transparent composite films were obtained and named **PDMS-PTC-355**, **PDMS-PTC-356**, **PDMS-PTC-357**, **PDMS-PTC-358**, and **PDMS-PTC-359** (Figure S24). The third-order NLO properties of these composite films were studied with a nanosecond laser using a typical open-hole Z-scanning system at 532 nm. We fixed the laser energy at 80 μJ, and the composite film thickness was controlled at 800 μm. The test results show that their transmittances are 0.80, 0.78, 0.49, 0.78, and 0.87, respectively (Table S1). The experimental results show that all composite films exhibit typical reverse saturation absorption (RSA) response, and from **PDMS-PTC-355** to **PDMS-PTC-359**, they exhibit successively enhanced optical limiting response (Figure 4a). At Z = 0, the minimum normalized transmission (Tmin) of **PDMS-PTC-355**, **PDMS-PTC-356**, **PDMS-PTC-357**, **PDMS-PTC-358,** and **PDMS-PTC-359** membranes are 0.82, 0.79, 0.70, 0.64 and 0.62, respectively (Figure 4a). To demonstrate the stability and homogeneity of these prepared films, we performed seven rounds of testing on the same **PDMS-PTC-359** film, but different test points were selected (Figure 4b). The results show that the Tmin of these different points are nearly identical. Figure 4d shows the change between the normalized transmittance of the samples and the laser input flux. It can be seen that the normalized transmittance decreases significantly with increasing laser energy, indicating that these samples have a significant nonlinear light limit effect.

In order to quantitatively evaluate the NLO performance of the sample, the nonlinear absorption coefficient of the sample was obtained from the open-hole Z-scan test results of the fitting test(β). Calculations of **PDMS-PTC-355**, **PDMS-PTC-356**, **PDMS-PTC-357**, **PDMS-PTC-358,** and **PDMS-PTC-359** composite films β Values are 2.5 × 10^−10^, 3.2 × 10^−10^, 6.5 × 10^−10^, 7.6 × 10^−10^ and 7.8 × 10^−10^ m/W (Figure 4c), respectively, which are obtained by comparison β. The values increase in turn. From Figure 4d, the OL values of the sample (Fol, the input flux whose transmittance is half of the linear transmittance) are 903.00 J/cm^2^(F_OL355_), 295.00 J/cm^2^(F_OL356_), 60.87 J/cm^2^(F_OL357_), 28.53 J/cm^2^(F_OL358_), 22.49 J/cm^2^(F_OL359_), respectively. Its OL value decreases in turn. Comparing the β Values and OL values show that **PDMS-PTC-359** has the highest β Value and the lowest OL value, indicating that its NLO performance is the highest. **PDMS-PTC-355** has the lowest β Value and the highest OL value, indicating that it has the lowest NLO performance. For better comparison, we also tested the third-order NLO properties of the starting material PDMS-PTC-101 (Zr) by the same method of film preparation. The result shows that it has no obvious optical limiting effect (Figure S25). Obviously, the self-assembly of anionic Zr_4_L_6_ cages with N, N-chelated transition-metal cations results in the activation and amplification of the NLO response. As described above, the increase in coordination bonds facilitates charge transfer between anion and cation groups, thus improving the third-order NLO performance. Herein, these assembled materials range from ion pair structure and dimer to 3,4-connected, 4-connected 3D frameworks; they have increasing coordinative interactions in turn, which may cause enhanced optical limiting effects.

## 3. Experimental Procedure

### 3.1. Materials and Methods

**Materials and instrumentation.** All reagents were purchased commercially and used without further purification. PTC-101(Zr), as the starting material of Zr_4_L_6_ cages, was massively synthesized by the method reported in our previous work [21]. Thermal gravimetric analyses (TGA) curves of these materials were carried out on a NETSCHZ STA-449C thermoanalyzer with a heating rate of 10 °C/min under a nitrogen atmosphere. Powder X-ray diffraction (XRD) patterns were recorded on a Rigaku Dmax/2500 X-ray diffractometer operating at 40 kV and 100 mA, using Cu-Kα or Ga-Kα radiation (λ = 1.54056 or 1.3405Å). The patterns were scanned over an angular range of 5–45° (2 theta) with a step length of 0.05° (2theta). The UV diffuse reflection data were recorded at room temperature using a powder sample with BaSO_4_ as a standard (100% reflectance) on a PerkinElmer Lamda-950 UV spectrophotometer. Fluorescence spectra were measured with a HORIBA Jobin-Yvon FluoroMax-4 spectrometer.

**X-ray Crystallography.** A high-quality single crystal was selected for the determination of single crystal structure. Crystallographic data of **PTC-355**–**PTC-359** were collected on a Supernova single crystal diffractometer equipped with graphite-monochromatic Cu-Kα or Ga-Kα radiation (λ = 1.54056 or 1.3405 Å) at 100 K. Absorption correction was applied using SADABS.^2^ Structure was solved by direct method and refined by full-matrix least-squares on F using SHELXTL. In these structures, some cations/anions and free guest molecules were highly disordered and could not be located. The diffused electron densities resulting from these residual cations/anions and guest molecules were removed from the data set using the SQUEEZE routine of PLATON and refined further using the data generated. Crystal data and details of data collection and refinement of **PTC-355**–**PTC-359** were summarized in Table 1 and Table 2. CCDC 2220530-2220534 contains the supplementary crystallographic data for this paper. These data are provided free of charge by The Cambridge Crystallographic Data Centre.

### 3.2. Synthesis of PTC-355-PTC-359

The simple method previously reported was used to synthesize large amounts of PTC-101(Zr) as the source of the Zr_4_L_6_ cages [21]. The specific synthesis method is as follows: Zr(O*n*Pr)_4_ (160 μL, 0.5 mmol), H_4_L (155 mg, 0.4 mmol), and 2 drops of ethylenediamine (en) were added to 6 mL of n-propanol/DMF (2:1, *v*/*v*) and mixed at room temperature (in order to improve the solubility of the reactive raw material, the mixture was treated with ultrasound). The above mixture was heated at 100 °C for 3 days. After cooling to room temperature, yellow polyhedral crystals of PTC-101(Zr) (Molecular formula: (Me_2_NH_2_)_8_[(Zr_4_L_6_)]·Guests) were obtained and then washed in the mother liquor. The final product was dried in the air and kept in the bottle. The yield of the final product was ~75% based on H_4_L.

**Synthesis of [(Zr_4_L_6_)(Cu(*trans*-DCH)_2_)_4_]·Guests (PTC-355).** PTC-101(Zr) (0.02 mmol, 80 mg), CuBr (0.12 mmol, 18 mg), and 8 drops of (+/−)-trans-1,2-diaminocyclohexane (*trans*-DCH) were dissolved to 6 mL of DMF/H_2_O (1:4, *v*/*v*) solvents and mixed at room temperature (In order to improve the solubility of the reactive raw material, the mixture was treated with ultrasound.). The above mixture was kept for 3 days at room temperature. Blue block crystals of **PTC-355** were produced and then washed and stored in the mother liquor. The yield of the final product was ~40% based on PTC-101(Zr).

**Synthesis of [(Zr_4_L_6_)(Zn(*trans*-DCH)_3_)_4_]·Guests (PTC-356).** PTC-101(Zr) (0.02 mmol, 80 mg), Zn(NO_3_)_2_·7H_2_O (0.18 mmol, 40 mg), and 2 drops of *trans*-DCH were dissolved in 6 mL of 1,4-dioxane/H_2_O/MeCN (2:2:2, *v*/*v*/*v*) solvents and mixed at room temperature (In order to improve the solubility of the reactive raw material, the mixture was treated with ultrasound.). The above mixture was heated at 80 °C for 3 days. After cooling to room temperature, yellow block crystals of **PTC-356** were produced and then washed and stored in the mother liquor. The yield of the final product was ~55% based on PTC-101(Zr).

**Synthesis of (Me_2_NH_2_)[(Zr_4_L_6_)(Zn_1/2_(*cis*-DCH))(Zn(*cis*-DCH)_3_)_2_]·Guests (PTC-357).** PTC-101(Zr) (0.02 mmol, 80 mg), Zn(CH_3_COO)_2_·2H_2_O (0.28 mmol, 60 mg), and 8 drops of *cis*-1,2-diaminocyclohexane (*cis*-DCH) were dissolved to 6 mL of DMF/H_2_O/MeCN (1:2:3, *v*/*v*/*v*) solvents and mixed at room temperature (In order to improve the solubility of the reactive raw material, the mixture was treated with ultrasound.). The above mixture was heated at 60 °C for 3 days. After cooling to room temperature, yellow block crystals of **PTC-357** were produced and then washed and stored in the mother liquor. The yield of the final product was ~45% based on PTC-101(Zr).

**Synthesis of [(Me_2_NH_2_)_8/3_[(Zr_4_L_6_)_5/6_(Zn(*cis*-DCH)_2_)]·Guests (PTC-358).** PTC-101(Zr) (0.02 mmol, 80 mg), ZnSO4·7H_2_O (0.09 mmol, 26 mg), and 2 drops of *cis*-DCH were dissolved to 6 mL of DMF/H_2_O/MeCN (1:2:3, *v*/*v*/*v*) solvents and mixed at room temperature (In order to improve the solubility of the reactive raw material, the mixture was treated with ultrasound.). The above mixture was heated at 60 °C for 3 days. After cooling to room temperature, yellow block crystals of **PTC-358** were produced and then washed and stored in the mother liquor. The yield of the final product was ~32% based on PTC-101(Zr).

**Synthesis of [(Me_2_NH_2_)_8_[(Zr_4_L_6_)_2_(Zn(*cis*-DCH)_2_)_4_]·Guests (PTC-359).** PTC-101(Zr) (0.02 mmol, 80 mg), Zn(CH_3_COO)_2_·2H_2_O (0.09 mmol, 20 mg), and 5 drops of *cis*-DCH were dissolved in 6 mL of DMF/H_2_O/MeCN (1:2:3, *v*/*v*/*v*) solvents and mixed at room temperature (In order to improve the solubility of the reactive raw material, the mixture was treated with ultrasound.). The above mixture was heated at 60 °C for 3 days. After cooling to room temperature, yellow block crystals of **PTC-359** were produced and then washed and stored in the mother liquor. The yield of the final product was ~ 50% based on PTC-101(Zr).

### 3.3. Manufacture Fabrication

**Manufacture of PTCs dispersed PDMS films.** PTCs refer to complex **PTC-355–PTC-359**. The crystals of **PTC-355**–**PTC-359** need to be dried in the air before sampling, respectively. First, 6 mg of the sample was mixed with 2 g PDMS (polydimethylsiloxane), and the sample was evenly dispersed by magnetic stirring for several hours. The second step is to add 1/10 mass of specific curing agent and continue to stir evenly for about 10 min. The third step is to take 1 g of the mixture and put it into a specific membrane. Under the action of gravity, the mixture is paved in the mold and then placed at room temperature for 0.5 h to eliminate bubbles. Finally, put the membrane utensil into a 60 °C oven for 5 h to obtain films for testing.

**Z-scan measurements.** The third-order NLO properties of the above sample were evaluated by using the Z-scan technique. The excitation light source was an Nd:YAG laser with a repetition rate of 5 Hz. The laser pulse (period, 5 ns; wavelength, 532 nm) was split into two beams with a mirror. The pulse energies at the front and back of the samples were monitored using energy detectors 1 and 2. All of the measurements were conducted at room temperature. The sample was mounted on a computer-controlled translation stage that shifted each sample along the z-axis.

**Calculation of the nonlinear optical parameters.** The relationship of the sample transmission and input fluence can be plotted from the open-aperture Z-scan curve. From the input laser pulse energy *E_in_* and beam radius *ω*(*z*), the light fluence *F_in_*(*z*) at any position can be obtained.

*F_in_*(*z*) is defined as:

$$F_{in}\left(z\right)=\frac{4E_{in}\sqrt{\ln2}}{\pi^{\frac{3}{2}}\;\omega \left(z\right){}^{2}}$$

where *ω*(*z*) is defined as:

$$\omega \left(z\right)=\frac{\omega_{0}}{{\left[1+{\left(\frac{z}{z_{0}}\right)}^{2}\right]}^{\frac{1}{2}}}$$

where *ω_0_* and *z*_0_ are the light beam radius and the Rayleigh range, respectively, and z_0_ is defined as:

$$z_{0}=\frac{k\omega_{0}^{2}}{2}$$

where *k* is defined as:

(1) $$k=\frac{2\pi }{\lambda }$$

## 4. Conclusions

In summary, we have shown that the combination of anionic Zr_4_L_6_ cages and N, N-chelated transition-metal cations can be used to prepare a series of interesting cage-based architectures, including ion pair structures (**PTC-355** and **PTC-356**), dimer (**PTC-357**), and 3D frameworks (**PTC-358** and **PTC-359**). Their single crystal structures have been well characterized and analyzed. Structural analyses show that **PTC-358** exhibits a 2-fold interpenetrating framework with a 3,4-connected topology, and **PTC-359** shows a 2-fold interpenetrating framework with a 4-connected **dia** network. In addition, powder XRD confirms that both **PTC-358** and **PTC-359** can be stable in air and other common solvents at room temperature. Furthermore, through a film-making method, we demonstrate that these materials, with increasing coordinative interactions in turn, have enhanced optical limiting effects, which is due to the high-efficiency charge transfer between anion and cation moieties. This work not only demonstrates an extraordinary cage-based assembly but also provides a series of promising materials for NLO applications.

## Figures and Tables

**Table 1:** Crystallographic Data and Structure Refinement Details for **PTC-355**–**PTC-357**.

Compounds	PTC-355	PTC-356	PTC-357
CCDC	2220530	2220531	2220532
Formula	C_198_H_218_Cu_4_N_20_O_44_Zr_4_	C_224_H_258_N_31_O_36_Zn_4_Zr_4_	C_180_H_172_N_16_O_41_Zn_2.5_Zr_4_
Formula weight	4200.95	4587.00	3743.63
Crystal system	monoclinic	trigonal	triclinic
Space group	*C*2/*c*	*R*3*c*	*P*-1
*a* (Å)	32.6048(5)	31.35400(10)	20.6382(4)
*b* (Å)	34.5994(5)	31.35400(10)	21.9542(4)
*c* (Å)	21.5330(2)	43.4738(2)	27.0262(6)
*α* (°)	90	90	77.715(2)
*β* (°)	96.0060(10)	90	83.131(2)
*γ* (°)	90	120	83.171(2)
*V* (Å^3^)	24,158.2(6)	37,012.1(3)	11,823.9(4)
*Z*	4	6	2
*D*_calcd_ (g cm^−3^)	1.155	1.235	1.052
*µ* (Cu/K*a*) (mm^−1^)	3.131	1.538	1.444
*F* (000)	8696.0	14,322.0	3854.0
Temperature (K)	100.15	100.00(10)	99.9(3)
Theta min, max (deg)	4.44, 97.712	4.528, 120.478	3.766, 109.766
Tot., uniq. Data	61,194, 17,465	69,417, 17,688	109,873, 41,094
Observed data [(*I* > 2*σ*(*I*)]	11,829	17,133	24,959
*R*_int_	0.0481	0.0221	0.0793
Data/restraints/parameters	17,465/368/1224	17,688/1/901	41,094/183/2199
*R*_1_, *wR*_2_ [*I* > 2*σ*(*I*)]	0.0939, 0.2518	0.0268, 0.0717	0.1278, 0.3346
*R*_1_, *wR*_2_ (all data)	0.1317, 0.2770	0.0279, 0.0723	0.1823, 0.3735
Goodness-of-fit on F^2^	1.033	1.044	1.042

**Table 2:** Crystallographic Data and Structure Refinement Details for **PTC-358** and **PTC-359**.

Compounds	PTC-358	PTC-359
CCDC	2220533	2220534
Formula	C_277.5_H_204_N_12_O_63_Zn_3_Zr_7_	C_338_H_283_N_23_O_73_Zn_4_Zr_8_
Formula weight	5549.22	6826.10
Crystal system	cubic	monoclinic
Space group	*I23*	*P2_1_/n*
*a* (Å)	42.5650(2)	29.0696(2)
*b* (Å)	42.5650(2)	42.3894(5)
*c* (Å)	42.5650(2)	37.7943(4)
*α* (°)	90	90
*β* (°)	90	92.5600(10)
*γ* (°)	90	90
*V* (Å^3^)	77,118.4(11)	46,525.3(8)
*Z*	8	4
*D*_calcd_ (g cm^−3^)	0.956	0.975
*µ* (Cu/K*a*) (mm^−1^)	1.431	1.395
*F* (000)	22,616.0	13,984.0
Temperature (K)	293(2)	99.9(3)
Theta min, max (deg)	4.42, 120.964	3.734, 120.576
Tot., uniq. Data	54,605, 24,502	103,020, 59,956
Observed data [(*I* > 2*σ*(*I*)]	18,321	371,508
*R*_int_	0.0198	0.0807
Data/restraints/parameters	24,502/205/1067	103,020/458/4023
*R*_1_, *wR*_2_ [*I* > 2*σ*(*I*)]	0.0574, 0.1743	0.1031, 0.2647
*R*_1_, *wR*_2_ (all data)	0.0723, 0.1864	0.1503, 0.2907
Goodness-of-fit on F^2^	1.077	1.019

## Data Availability

Not applicable.

## Supplementary Materials

The following supporting information can be downloaded at: https://www.mdpi.com/article/10.3390/molecules28052301/s1, Figure S1. The asymmetric unit of **PTC-355**, showing half of Zr_4_L_6_ cage, two [Cu(transDCH)_2_]^2+^ cations, and some solvent molecules. (Other solvents could not be located because of highly disorder); Figure S2. The packed structure along the c-axis and the supramolecular interactions in **PTC-355**; Figure S3. The asymmetric unit of **PTC-356**, showing one third of Zr_4_L_6_ cage, one and one third of [Zn(trans-DCH)_3_]^2+^ cations, and some solvent molecules. (Other solvents could not be located because of highly disorder); Figure S4. The asymmetric unit of **PTC-357**, showing one Zr_4_L_6_ cage, half of [Zn(cisDCH)_2_]^2+^ and two [Zn(cis-DCH)_3_]^2+^cations, one (Me_2_NH_2_)^+^ cation and some solvent molecules. (Some cis-DCH and other solvents could not be located because of highly disorder); Figure S5. The packed structure of **PTC-357**; Figure S6. The asymmetric unit of **PTC-358**, showing 5/6 Zr4L6 cage and one [Zn(cisDCH)_2_]^2+^ cation. ((Me_2_NH_2_)^+^ cations and solvents could not be located because of highly disorder); Figure S7. The asymmetric unit of **PTC-359**, showing two Zr_4_L_6_ cages, four [Zn(cisDCH)_2_]^2+^ cations, one ((Me_2_NH_2_)^+^ cation and some solvent molecules. (Other cations and solvents could not be located because of highly disorder); Figure S8. TGA curve of **PTC-355**; Figure S9. TGA curve of **PTC-356**; Figure S10. TGA curve of **PTC-357**; Figure S11. TGA curve of **PTC-358**; Figure S12. TGA curve of **PTC-359**; Figure S13. (red); Figure S14. PXRD patterns of simulated from the single-crystal data of **PTC-356** (black) and as-synthesized **PTC-356** (red); Figure S15. PXRD patterns of simulated from the single-crystal data of **PTC-357** (black) and as-synthesized **PTC-357** (red); Figure S16. The excitation spectrum of L (embonate) ligand; Figure S17. The emission spectrum of L (embonate) ligand; Figure S18. The excitation spectrum of compound **PTC-101**(Zr); Figure S19. The excitation spectrum of **PTC-355**; Figure S20. The excitation spectrum of **PTC-356**; Figure S21. The excitation spectrum of **PTC-357**; Figure S22. The excitation spectrum of **PTC-358**; Figure S23. The excitation spectrum of **PTC-359**; Figure S24. The photos of PDMS-PTCs films (PTCs refers to compounds **PTC-355** to **PTC359**; Figure S25. OA Z-scan (points) and theoretical fit (solid lines) curve of PDMS-PTC-101(Zr); Figure S26. The frontier molecular orbitals of **PTC-359**, which was obtained from the DFT Calculations; Table S1. Linear and NLO data of PDMS-PTCs films.
