# Supplementary material for: Enhancing Third-Order Nonlinear Optical Property by Regulating Interaction between Zr4(embonate)6 Cage and N, N-Chelated Transition-Metal Cation
Source: Molecules. 2023 Mar 1;28(5):2301. doi: 10.3390/molecules28052301 (PMC10005618; doi:10.3390/molecules28052301)
Supplement: Supplementary file 1 [file molecules-28-02301-s001.zip › molecules-2223840-supplementary.pdf]

Supplementary Materials

Enhancing Third-Order Nonlinear Optical Property by Regulating Interaction between Zr_4(embonate)$_6$ Cage and N,
N-Chelated Transition-Metal Cation

Gang Xiang [1,2,†], Na Li [1,2,†], Guang-Hui Chen [2], Qiao-Hong Li [2], Shu-Mei Chen [1,*], Yan-Ping He [2,*] and Jian Zhang [2]

[1] College of Chemistry, Fuzhou University, Fuzhou 350108, China; xianggang309@163.com (G.X.); lina@fjirsm.ac.cn (N.L.)
[2] State Key Laboratory of Structural Chemistry, Fujian Institute of Research on the Structure of Matter,
Chinese Academy of Sciences, Fuzhou 350002, China; chenguanghui@fjirsm.ac.cn (G.-H.C.); lqh2382@fjirsm.ac.cn (Q.-H.L.); zhj@fjirsm.ac.cn (J.Z.)
* Correspondence: csm@fzu.edu.cn (S.-M.C.); hyp041@163.com (Y.-P.H.)
† These authors contributed equally to this work.

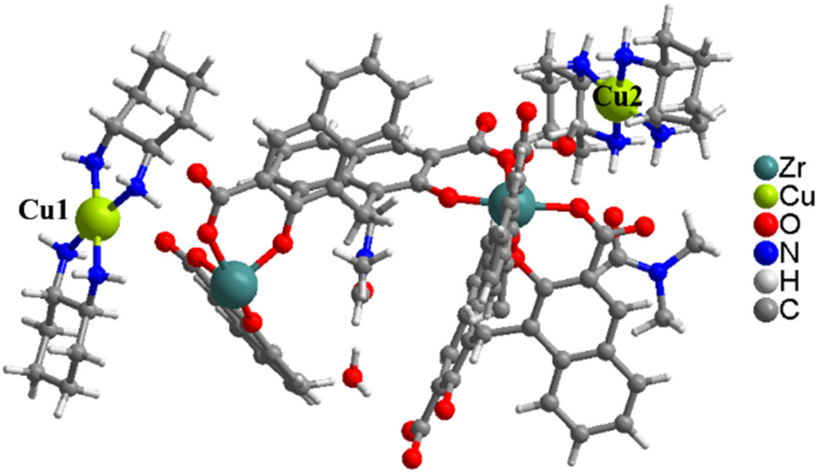

Figure S1. The asymmetric unit of **PTC-355**, showing half of Zr_4L_6 cage, two [Cu(*trans*-DCH)$_2$]$^{2+}$ cations, and some solvent molecules. (Other solvents could not be located because of highly disorder).

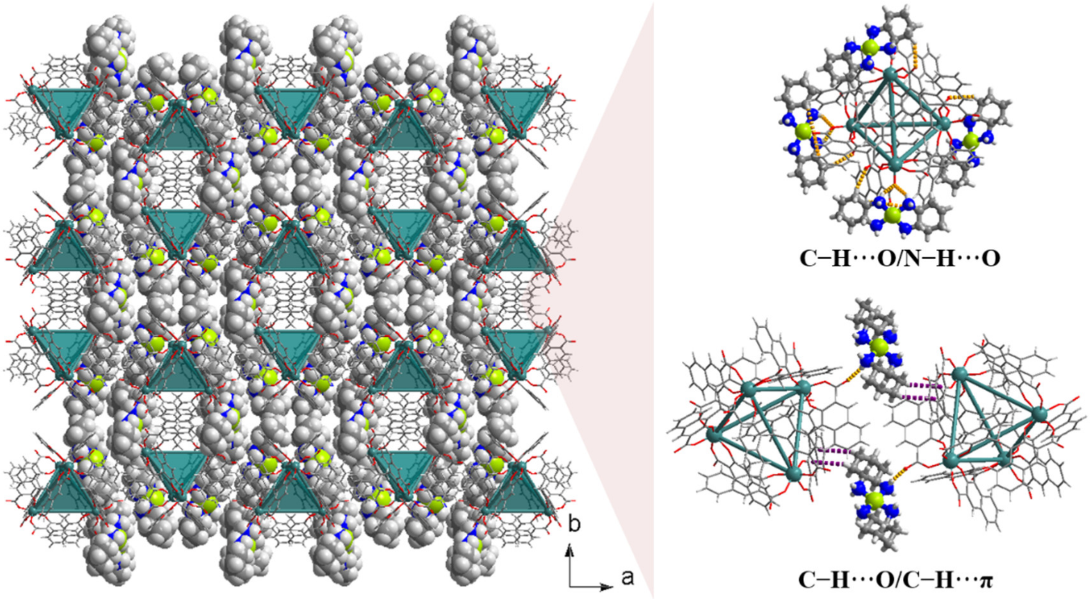

Figure S2. The packed structure along the c-axis and the supramolecular interactions in **PTC-355**.

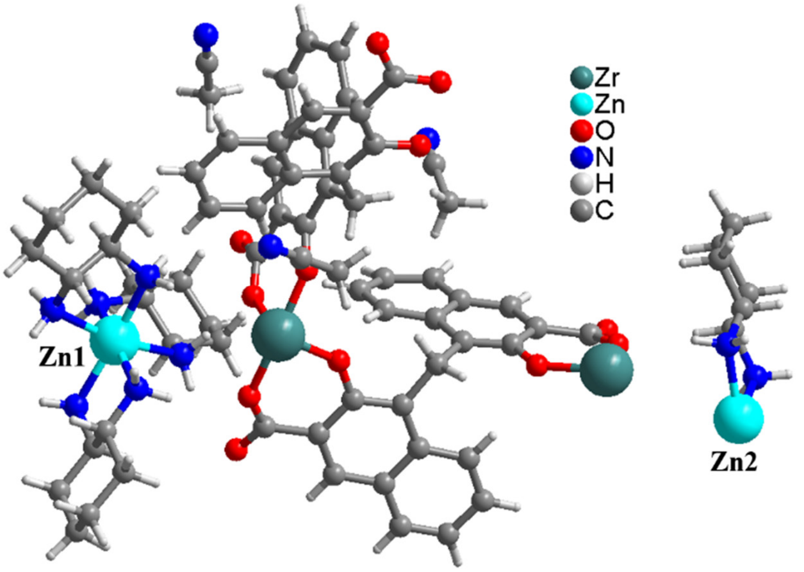

Figure S3. The asymmetric unit of **PTC-356**, showing one third of Zr_4L_6 cage, one and one third of $[Zn(\textit{trans}\text{-DCH})_3]^{2+}$ cations, and some solvent molecules. (Other solvents could not be located because of highly disorder).

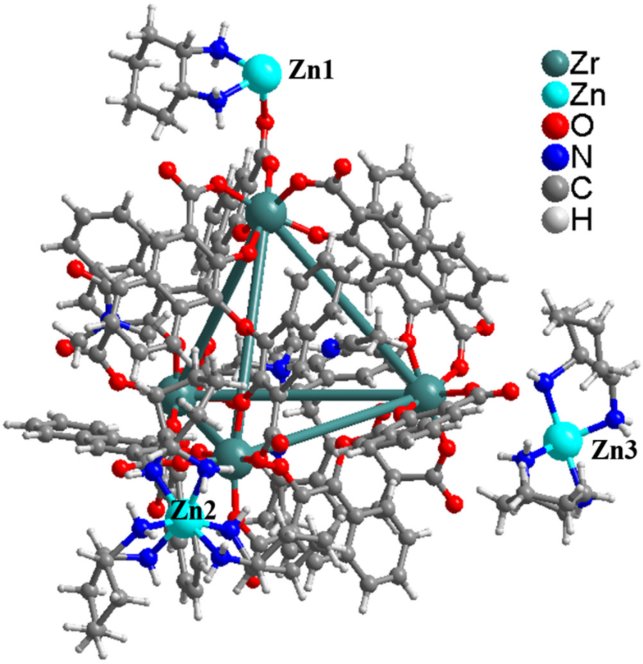

Figure S4. The asymmetric unit of **PTC-357**, showing one Zr_4L_6 cage, half of $[Zn(\textit{cis}\text{-}DCH)_2]^{2+}$ and two $[Zn(\textit{cis}\text{-}DCH)_3]^{2+}$ cations, one $(Me_2NH_2)^+$ cation and some solvent molecules. (Some *cis*-DCH and other solvents could not be located because of highly disorder).

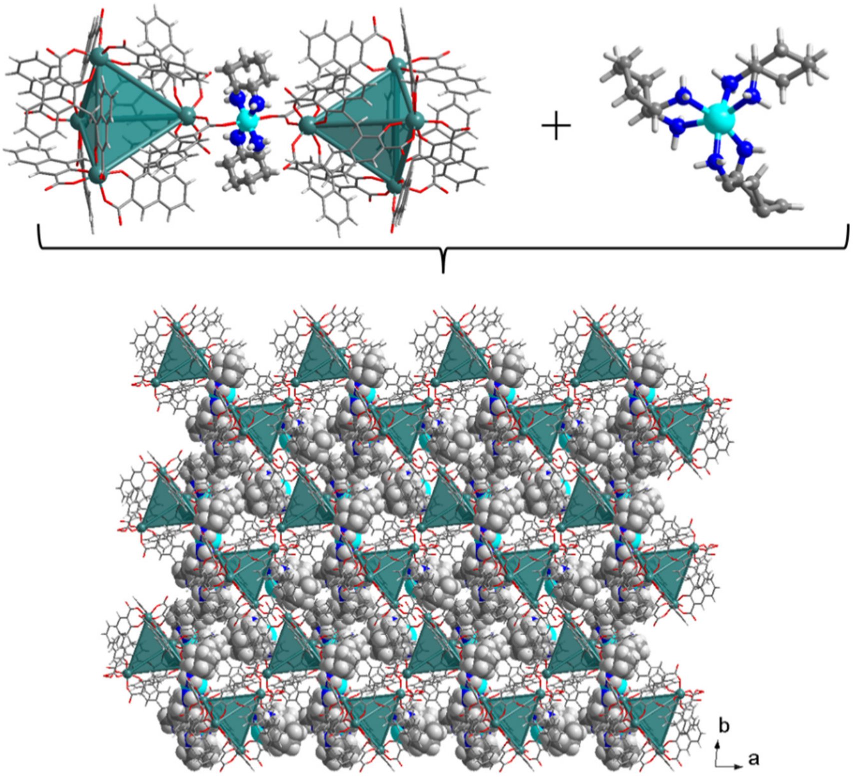

Figure S5. The packed structure of **PTC-357**.

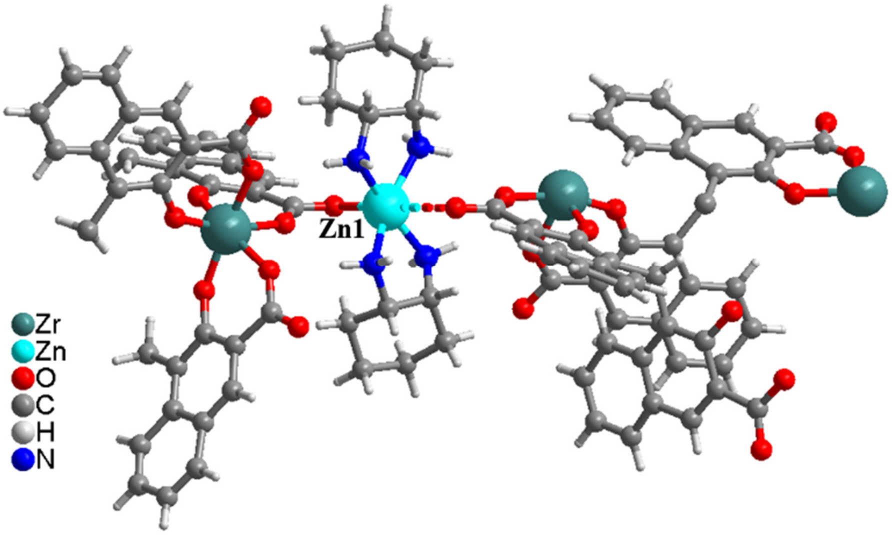

Figure S6. The asymmetric unit of **PTC-358**, showing 5/6 Zr_4L_6 cage and one $[Zn(\textit{cis}\text{-}DCH)_2]^{2+}$ cation. ($(Me_2NH_2)^+$ cations and solvents could not be located because of highly disorder).

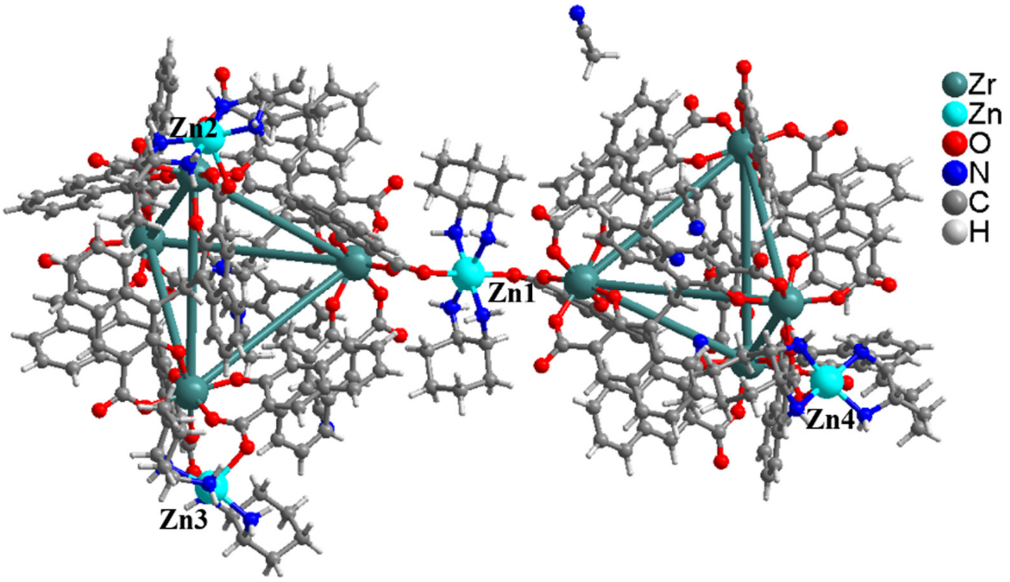

Figure S7. The asymmetric unit of **PTC-359**, showing two Zr_4L_6 cages, four $[Zn(\textit{cis}\text{-}DCH)_2]^{2+}$ cations, one $(Me_2NH_2)^+$ cation and some solvent molecules. (Other cations and solvents could not be located because of highly disorder).

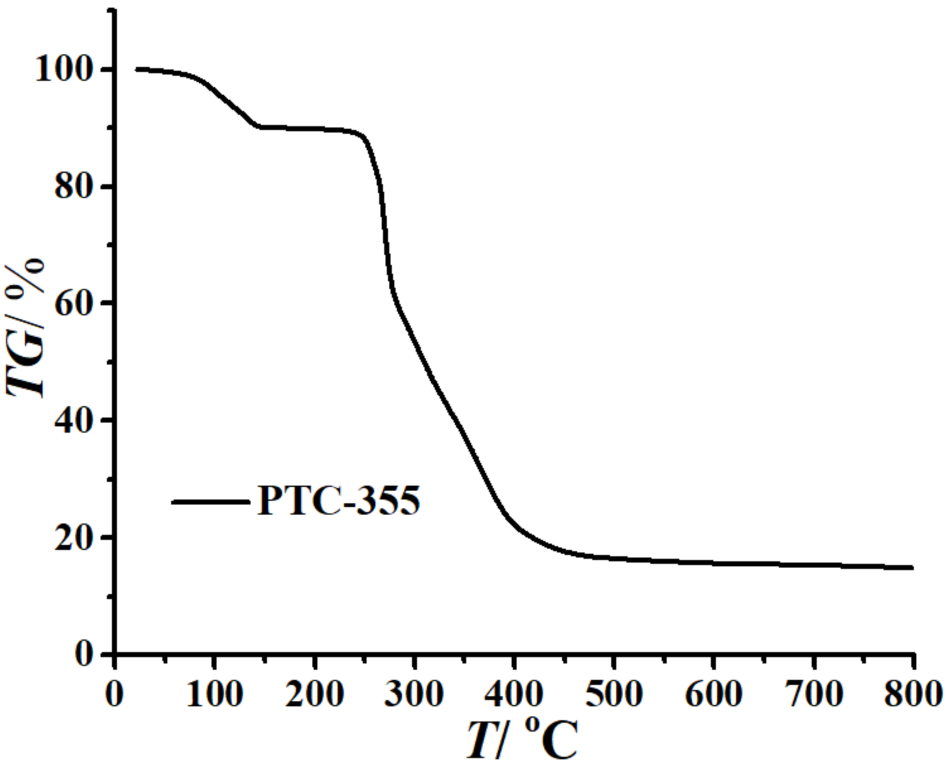

Figure S8. TGA curve of **PTC-355**.

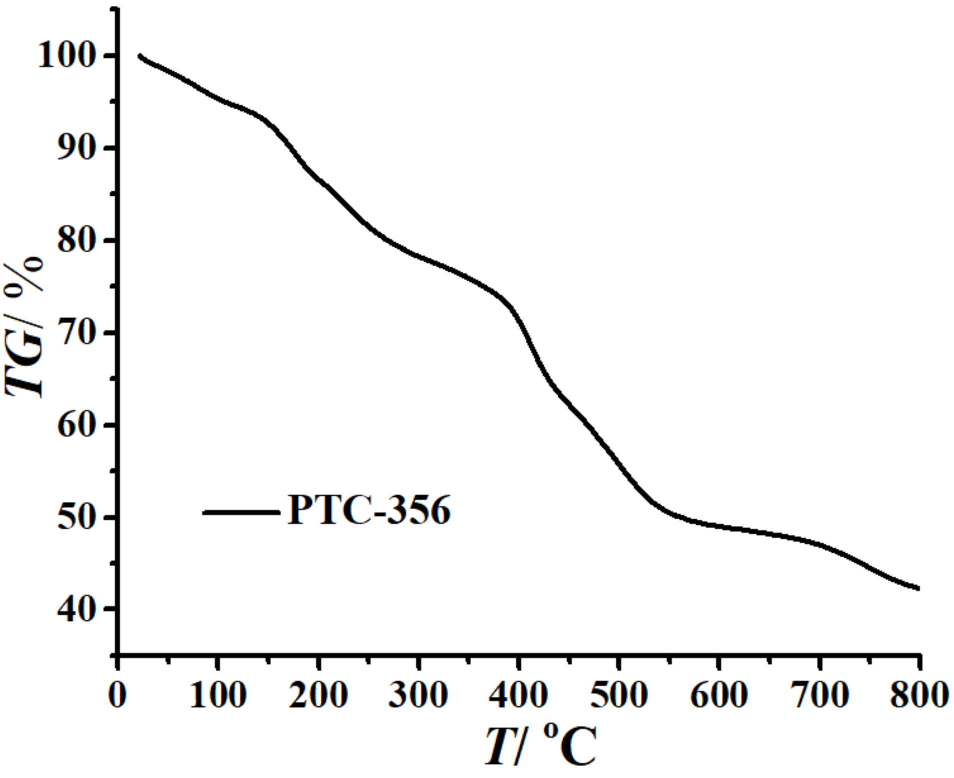

Figure S9. TGA curve of **PTC-356**.

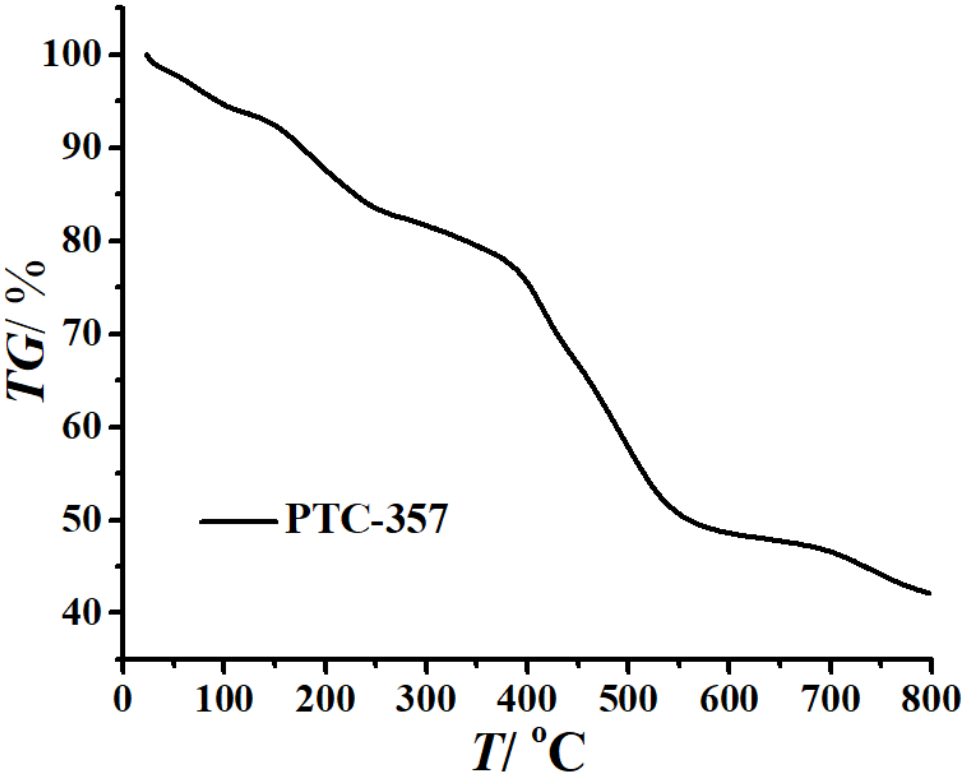

Figure S10. TGA curve of **PTC-357**.

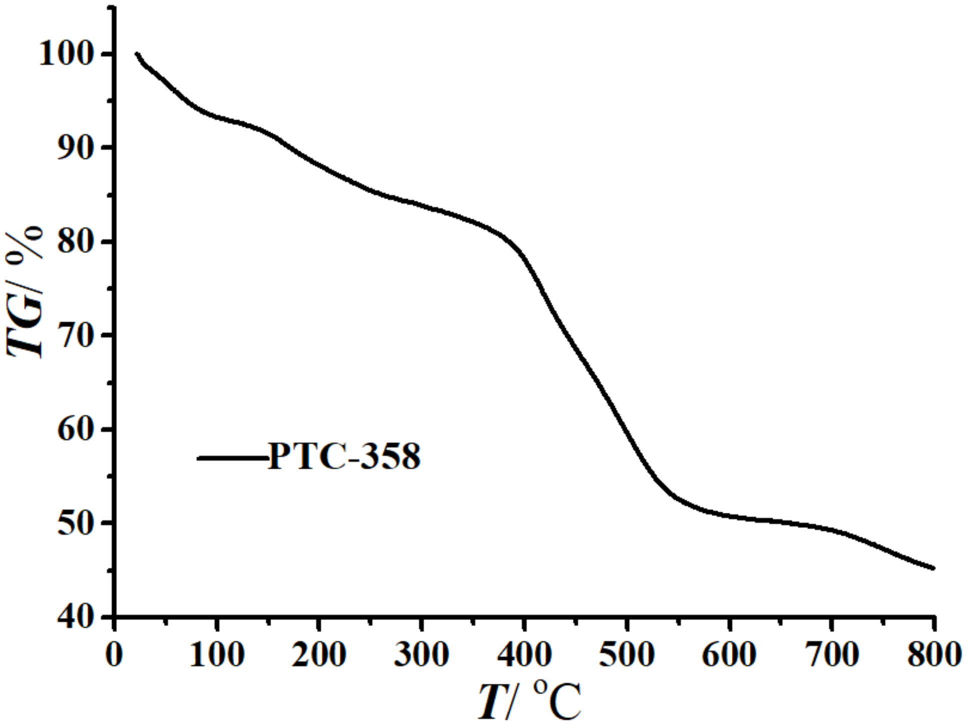

Figure S11. TGA curve of **PTC-358**.

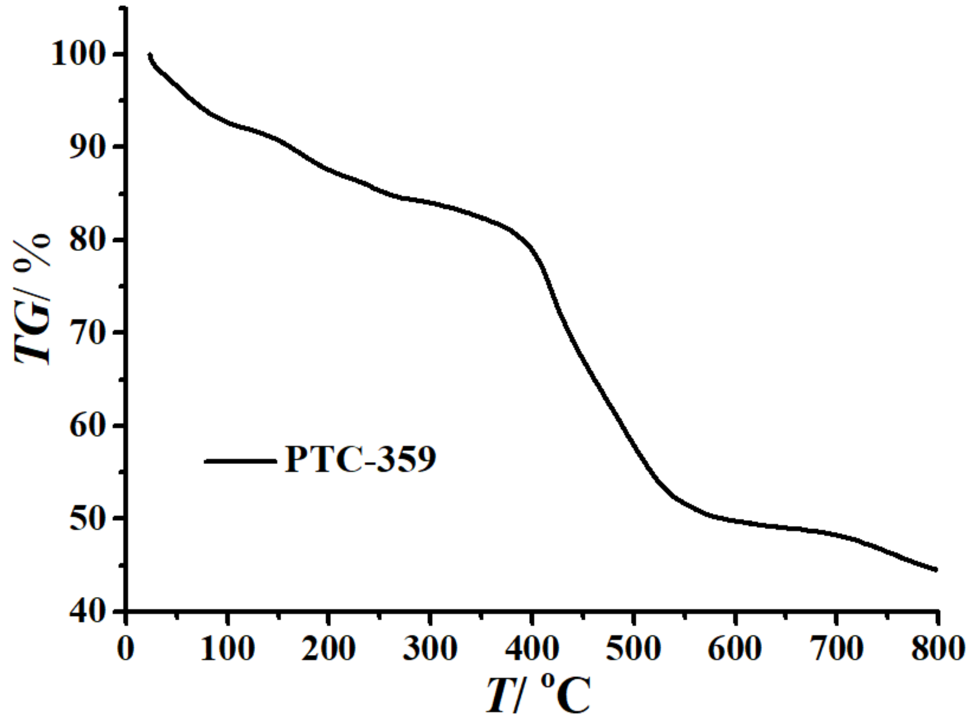

Figure S12. TGA curve of **PTC-359**.

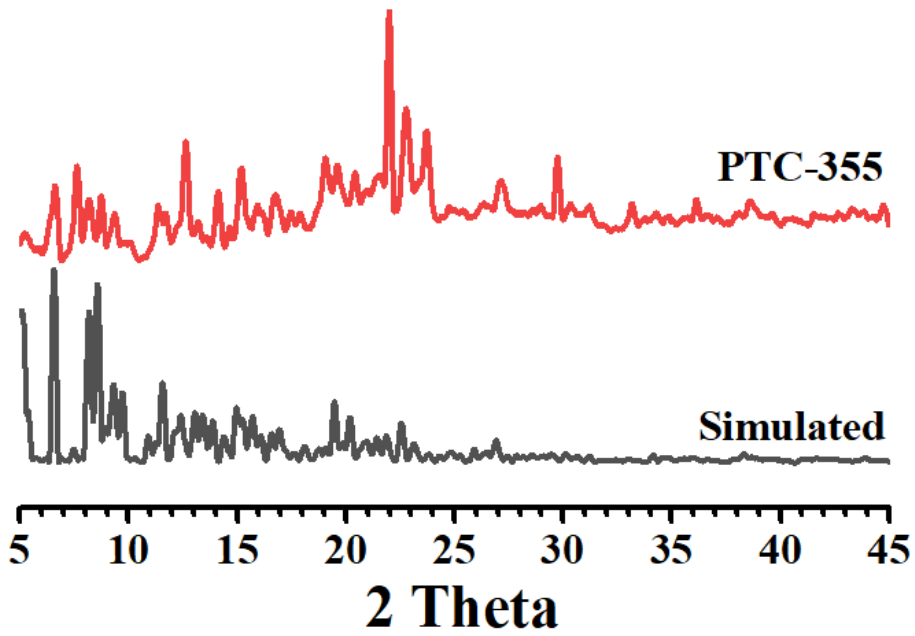

Figure S13. PXRD patterns of simulated from the single-crystal data of **PTC-355**(black) and as-synthesized **PTC-355** (red).

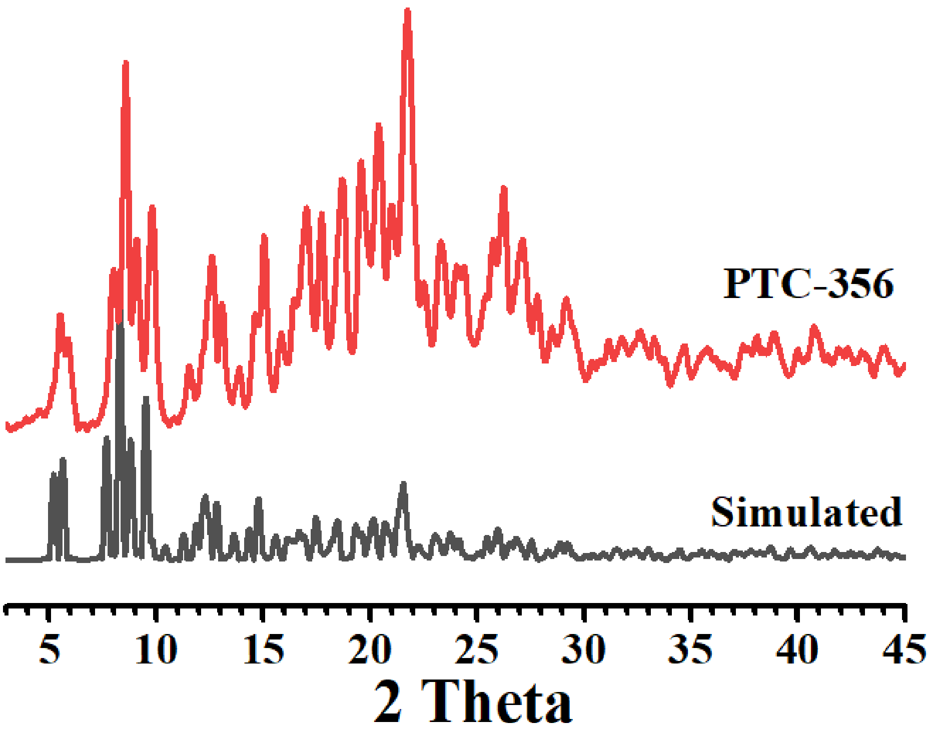

Figure S14. PXRD patterns of simulated from the single-crystal data of **PTC-356** (black) and as-synthesized **PTC-356** (red).

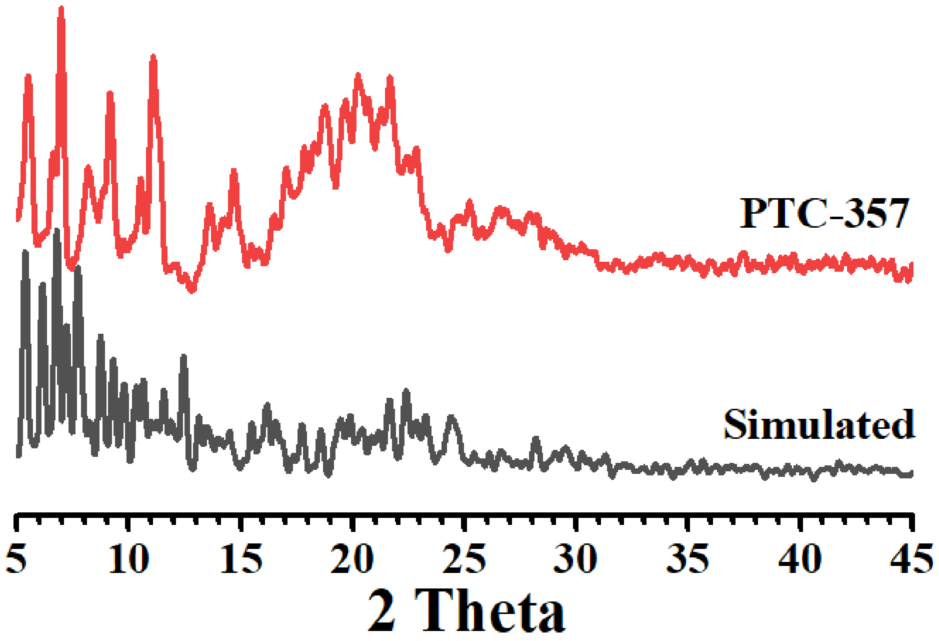

Figure S15. PXRD patterns of simulated from the single-crystal data of **PTC-357** (black) and as-synthesized **PTC-357** (red).

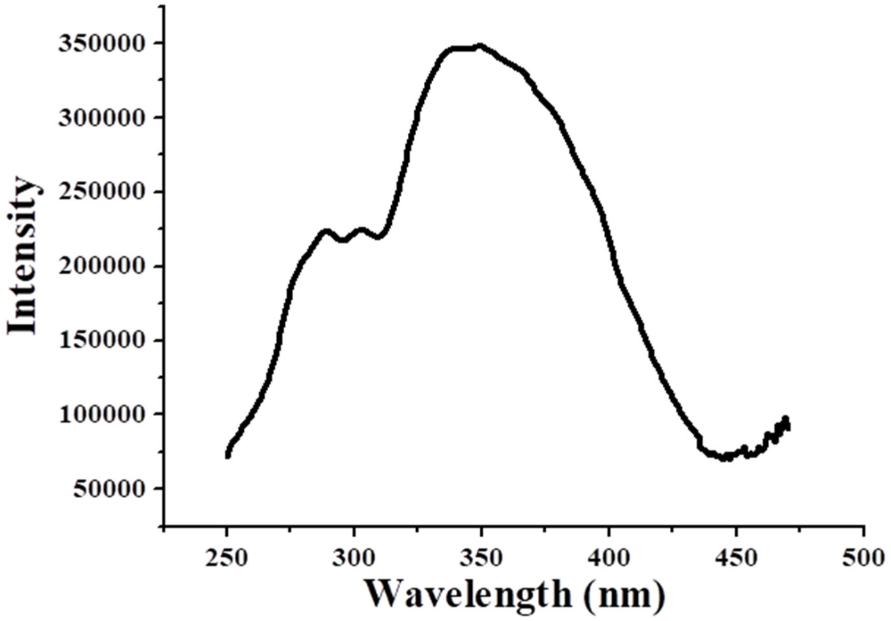

Figure S16. The excitation spectrum of L (embonate) ligand.

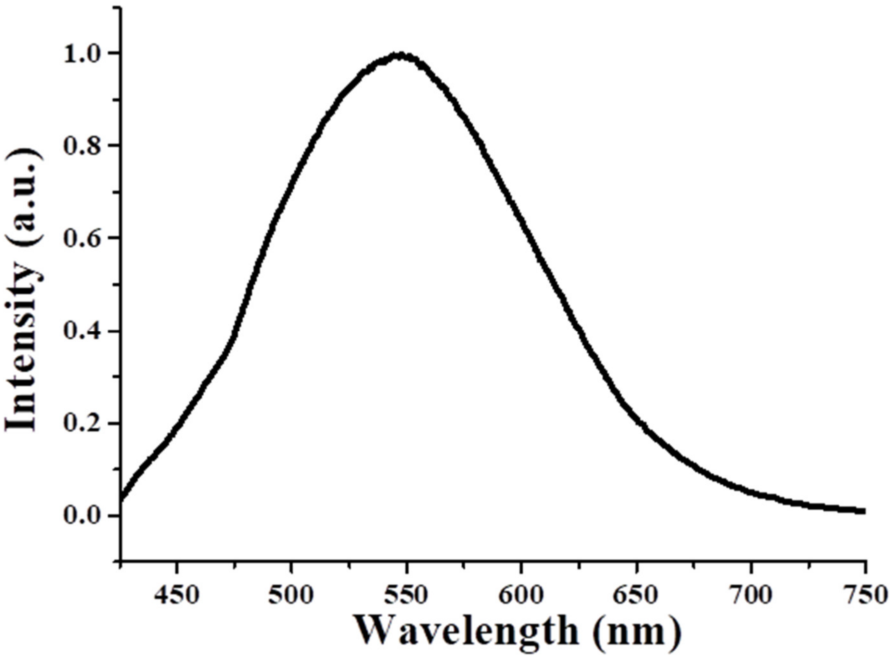

Figure S17. The emission spectrum of L (embonate) ligand.

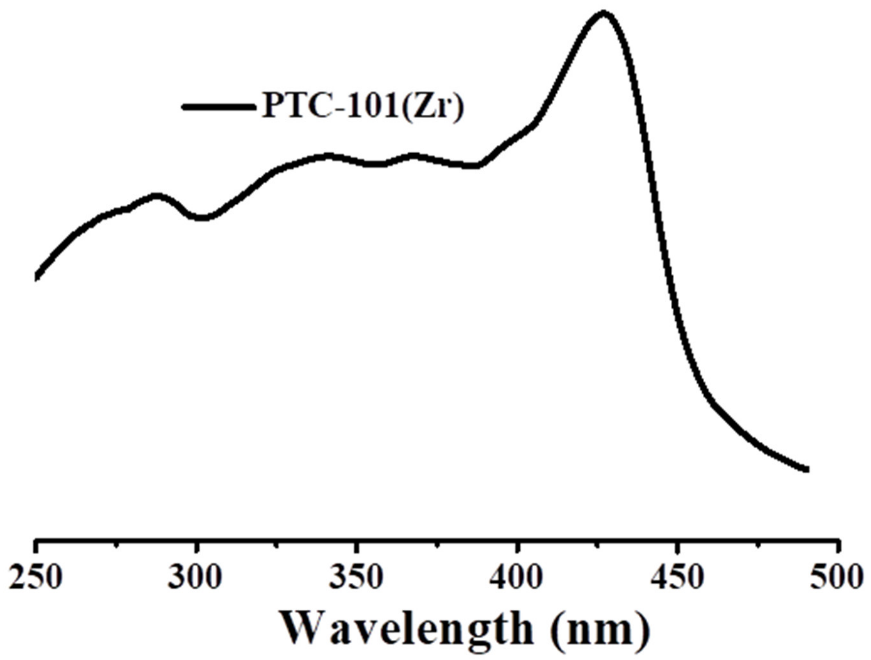

Figure S18. The excitation spectrum of compound **PTC-101(Zr)**.

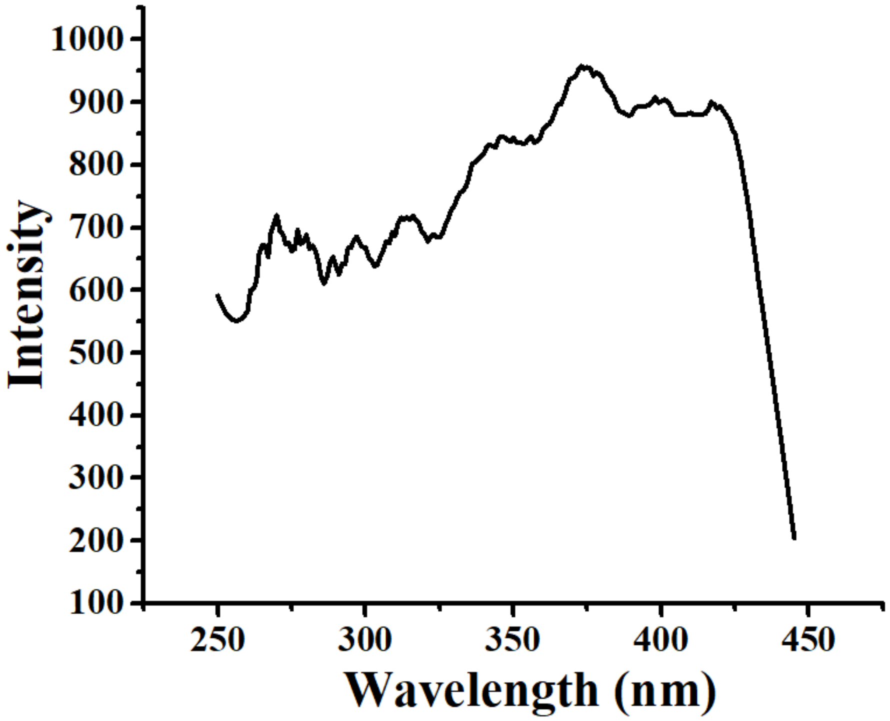

Figure S19. The excitation spectrum of **PTC-355**.

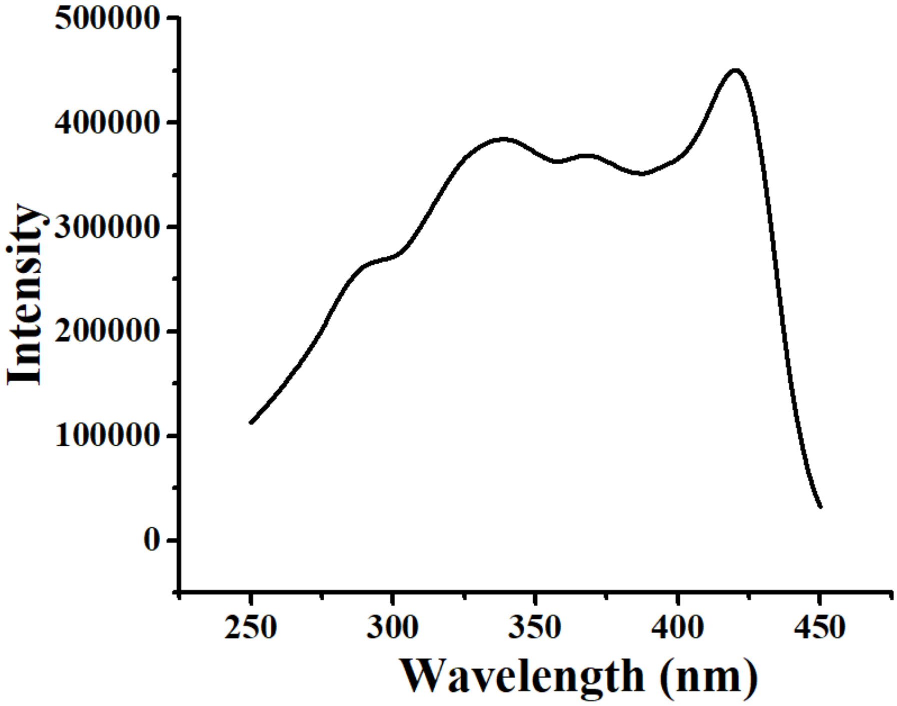

Figure S20. The excitation spectrum of **PTC-356**.

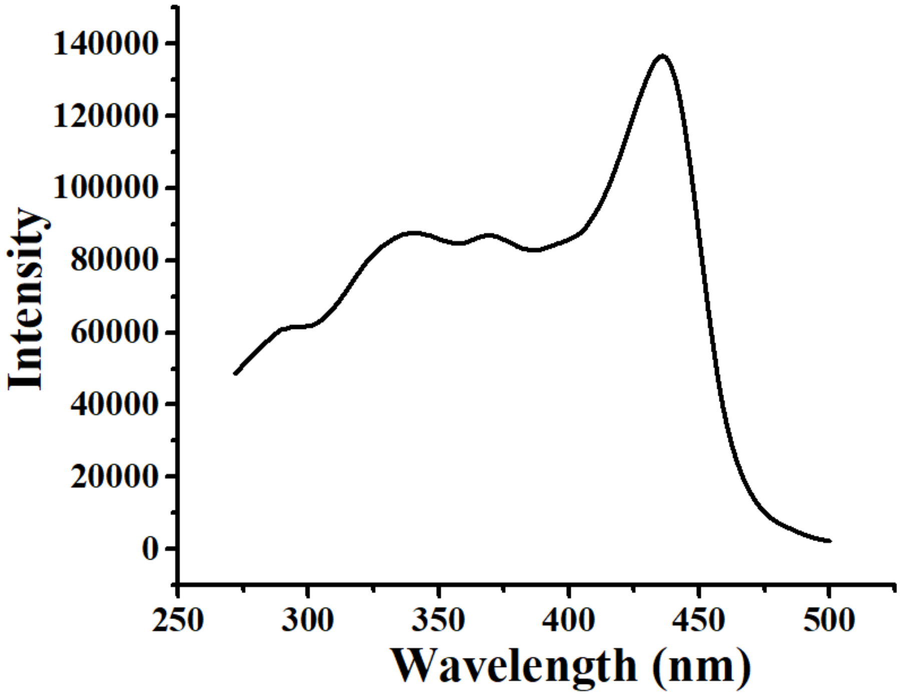

Figure S21. The excitation spectrum of **PTC-357**.

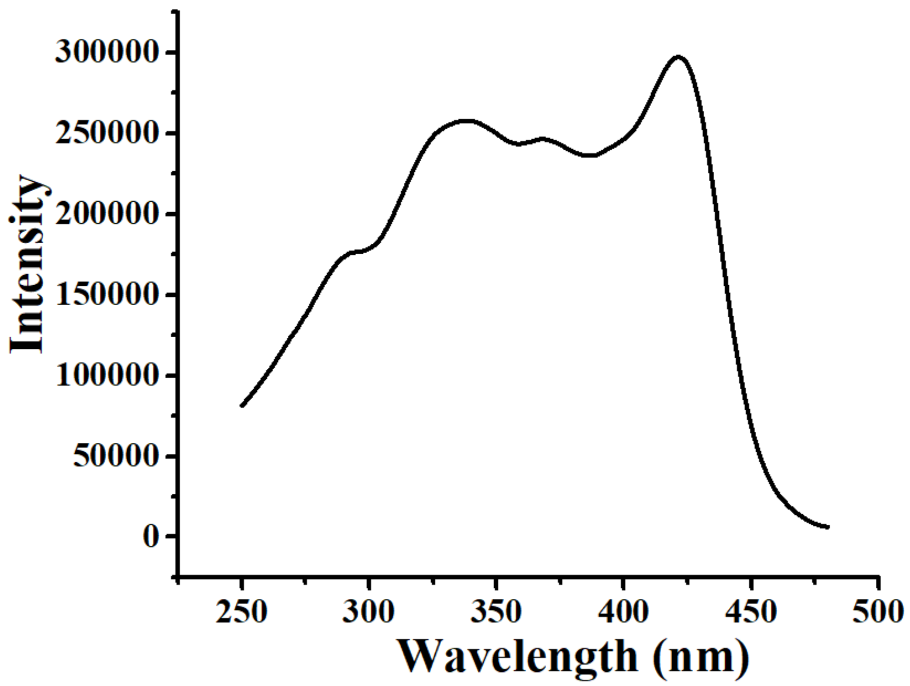

Figure S22. The excitation spectrum of **PTC-358**.

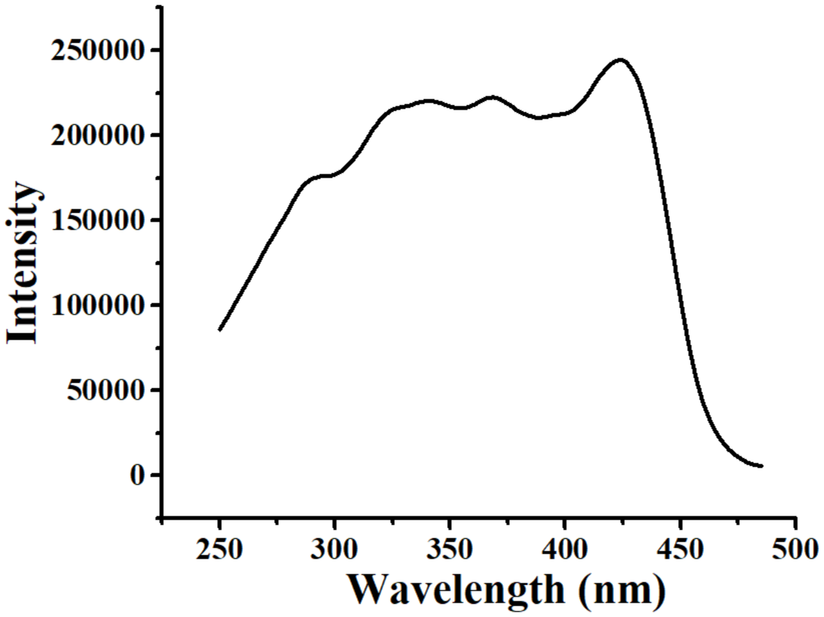

Figure S23. The excitation spectrum of **PTC-359**.

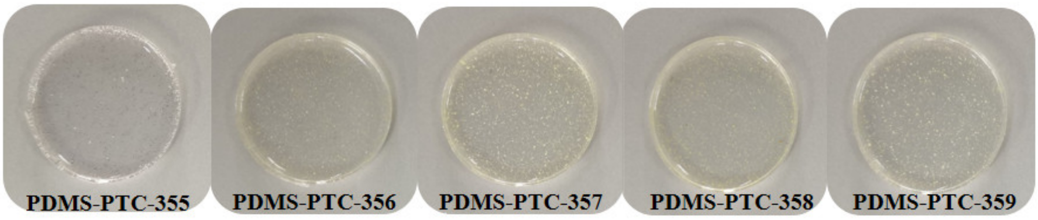

Figure S24. The photos of PDMS-PTCs films (PTCs refers to compounds **PTC-355** to **PTC-359**.

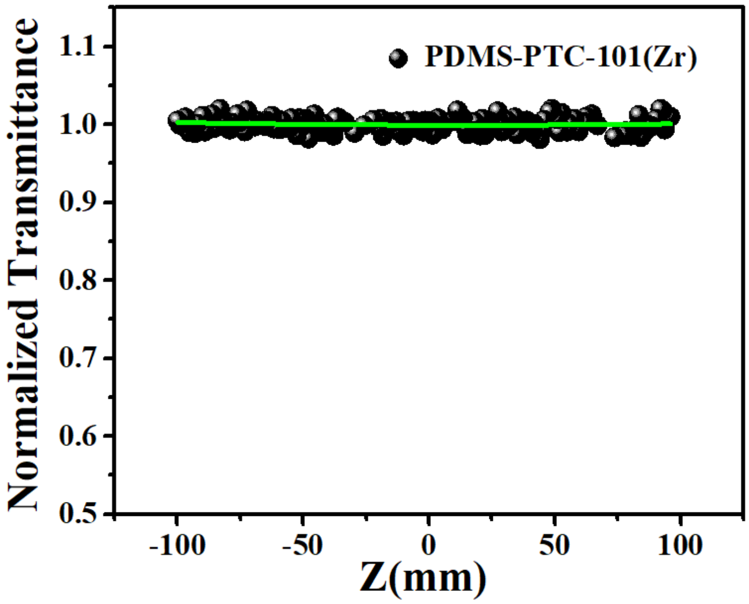

Figure S25. OA Z-scan (points) and theoretical fit (solid lines) curve of PDMS-PTC-101(Zr).

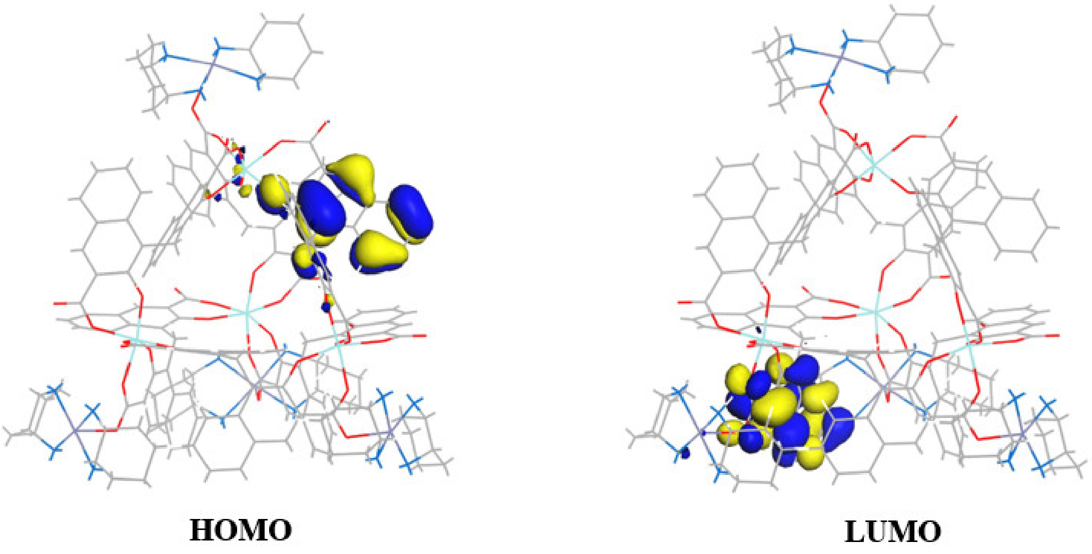

Figure S26. The frontier molecular orbitals of **PTC-359**, which was obtained from the DFT calculations.

Table S1. Linear and NLO data of PDMS-PTCs films.

Samples	PDMS-PTC-355	PDMS-PTC-356	PDMS-PTC-357	PDMS-PTC-358	PDMS-PTC-359
**Energy(μJ)**	80	80	80	80	80
**Thickness(μm)**	800	800	800	800	800
**Tmin**	0.82	0.79	0.70	0.64	0.62
**T_0**	0.80	0.78	0.49	0.78	0.87
**β (*10^{-10}m/W)**	2.5	3.2	6.5	7.6	7.8

Energy: Energy passing through the film, Thickness: thickness of film, Tmin: the minimum normalized transmission: T_0: linear transmittance; β: nonlinear coefficient.
